# Supplementary material for: Quadriceps muscle reaction time in obese children
Source: PeerJ. 2024 Feb 29;12:e17050. doi: 10.7717/peerj.17050 (PMC10909349; doi:10.7717/peerj.17050)
Supplement: Supplemental Information 3 [file peerj-12-17050-s003.docx]

STROBE Statement—checklist of items that should be included in reports of observational studies

|  | Item No. | Recommendation | Page  No. | Relevant text from manuscript |
| --- | --- | --- | --- | --- |
| **Title and abstract** | 1 | (*a*) Indicate the study’s design with a commonly used term in the title or the abstract | 1 | “The study utilized a cross-sectional design” |
|  |  | (*b*) Provide in the abstract an informative and balanced summary of what was done and what was found | 1 | “The sample size consisted of 42 schoolchildren (54.5% girls) aged 11 to 12 years old. Participant measurements included weight and height, which were used to categorize individuals based on BMI. Additionally, the electrical bioimpedance technique was employed to categorize participants based on their body fat percentage. A sudden destabilization test of the lower limb was performed to assess the reaction time of the rectus femoris, vastus medialis, and vastus lateralis muscles. The results show that overweight/obese children have a longer muscle reaction time for both the rectus femoris (β = 18.13; *p* = 0.048) and the vastus lateralis (β = 14.51; *p* = 0.042). Likewise, when the children were classified by percentage of body fat the results showed that overfat/obese children have a longer muscle reaction time for both the rectus femoris (β = 18.13; p = 0.048) and the vastus lateralis (β = 14.51; p = 0.042).” |
| Introduction | | | |  |
| Background/rationale | 2 | Explain the scientific background and rationale for the investigation being reported | 2-3 | The introduction highlights the epidemiology of childhood obesity, the motor consequences and the possible causes of functional disorders due to obesity. |
| Objectives | 3 | State specific objectives, including any prespecified hypotheses | 3 | “Therefore, our study aimed to determine the influence of nutritional status, according to body mass index (BMI) and fat mass percentage, on quadriceps muscle reaction times. We hypothesize that those categorized as overweight/obese and overfat/obese will have delayed muscle reaction times” |
| Methods | | | |  |
| Study design | 4 | Present key elements of study design early in the paper | 3 | “The study utilized a cross-sectional design” |
| Setting | 5 | Describe the setting, locations, and relevant dates, including periods of recruitment, exposure, follow-up, and data collection | 3 | “The participants were evaluated in a 30-minute session in a room at 21ºC in the presence of their parents and/or guardians. Participants wore shorts and were barefoot during the tests. The evaluations were performed on nutritional status and muscular reaction time.” |
| Participants | 6 | (*a*) *Cohort study*—Give the eligibility criteria, and the sources and methods of selection of participants. Describe methods of follow-up  *Case-control study*—Give the eligibility criteria, and the sources and methods of case ascertainment and control selection. Give the rationale for the choice of cases and controls  *Cross-sectional study*—Give the eligibility criteria, and the sources and methods of selection of participants | 3-4 | “The sample size consisted of 42 schoolchildren (54.5% girls) aged 11 to 12 years old (girls, age: 11.55 ± 0.41 years; body mass: 47.75 ± 11.57 kg; height: 1.48 ± 0.05 m; and boys, age: 11.61 ± 0.44 years; body mass: 44.62 ± 8.69 kg; height: 1.45 ± 0.05 m), who attended a public educational institution in the city of Maule, Chile. Participants were selected under a non-probabilistic convenience sampling. The exclusion criteria for participants were as follows: (a) individuals displaying neurological abnormalities, (b) those who experienced musculoskeletal injuries in the lower limb, including fractures, sprains, dislocations, or muscle tears within six months before the assessments, (c) the presence of any inflammatory or painful conditions during the assessments in the lower limb, and (d) dependence on walking aids or assistive devices.” |
|  |  | (*b*) *Cohort study*—For matched studies, give matching criteria and number of exposed and unexposed  *Case-control study*—For matched studies, give matching criteria and the number of controls per case |  |  |
| Variables | 7 | Clearly define all outcomes, exposures, predictors, potential confounders, and effect modifiers. Give diagnostic criteria, if applicable | 4-5 | It is indicated through subtitles, stating the procedures in detail. |
| Data sources/ measurement | 8* | For each variable of interest, give sources of data and details of methods of assessment (measurement). Describe comparability of assessment methods if there is more than one group | 4-5 | It is indicated through subtitles, stating the procedures in detail. |
| Bias | 9 | Describe any efforts to address potential sources of bias | 4-5 | They are implicit in the methodology of the manuscript. |
| Study size | 10 | Explain how the study size was arrived at | 4 | “The sample size was determined based on the mean difference in the amplitude of the electromyographic signal of the rectus femoris muscle, as observed in a comparative study involving adolescents of normal weight and those who are obese while walking (De Carvalho et al., 2012). The study suggests a substantial mean difference of 10.56% between these two groups. Utilizing this information, the sample size for the current research was computed to encompass 40 participants. This calculation incorporated a significance level of 0.1 and a statistical power of 85%, ensuring the study's ability to detect meaningful effects.  “ |

Continued on next page

| Quantitative variables | 11 | Explain how quantitative variables were handled in the analyses. If applicable, describe which groupings were chosen and why | 5 | They are declared when explaining the registration of each variable. |
| --- | --- | --- | --- | --- |
| Statistical methods | 12 | (*a*) Describe all statistical methods, including those used to control for confounding | 5 | “Data were analyzed with Graph Pad Prism 9.0 statistical software (GraphPad Software, La Jolla, CA). The data of the studied sample are presented as mean and standard deviation for continuous variables and as percentages for categorical variables. The Shapiro-Wilk test was performed to determine the distribution of the data. To compare muscle reaction times according to BMI and fat mass percentage, the t-student test was used for independent samples. A multiple linear regression model (95% confidence interval) was applied. The dependent variable was muscle reaction time, while the independent variables were nutritional status and sex (boys and girls). The regression models were made separately according to two ways of classifying nutritional status: (a) according to the classification by BMI (normal-weight and overweight/obese); (b) according to the classification by fat mass percentage (normal and overfat/obese). Two regression models were generated; model 1 included nutritional status and sex, while model 2 only considered nutritional status. The goodness of fit was assessed using the R^2^ coefficient. A collinearity diagnosis was conducted for each variable included in the regression models. Variables with tolerance values less than 0.10 and variance inflation factor (VIF) values exceeding 10.0 were eliminated. The level of significance for all statistical tests was set at <0.05.  “ |
|  |  | (*b*) Describe any methods used to examine subgroups and interactions |  |  |
|  |  | (*c*) Explain how missing data were addressed |  |  |
|  |  | (*d*) *Cohort study*—If applicable, explain how loss to follow-up was addressed  *Case-control study*—If applicable, explain how matching of cases and controls was addressed  *Cross-sectional study*—If applicable, describe analytical methods taking account of sampling strategy |  |  |
|  |  | (*e*) Describe any sensitivity analyses |  |  |
| Results | | | | |
| Participants | 13* | (a) Report numbers of individuals at each stage of study—eg numbers potentially eligible, examined for eligibility, confirmed eligible, included in the study, completing follow-up, and analysed | 6 | “Forty-two children participated in the study (54.5% girls), 57.1% were classified as overweight/obese according to BMI, and 42.9% were classified as overfat/obese according to fat mass percentage.” |
|  |  | (b) Give reasons for non-participation at each stage |  |  |
|  |  | (c) Consider use of a flow diagram |  |  |
| Descriptive data | 14* | (a) Give characteristics of study participants (eg demographic, clinical, social) and information on exposures and potential confounders | 3 | “42 schoolchildren (54.5% girls) aged 11 to 12 years old (girls, age: 11.55 ± 0.41 years; body mass: 47.75 ± 11.57 kg; height: 1.48 ± 0.05 m; and boys, age: 11.61 ± 0.44 years; body mass: 44.62 ± 8.69 kg; height: 1.45 ± 0.05 m)” |
|  |  | (b) Indicate number of participants with missing data for each variable of interest |  |  |
|  |  | (c) *Cohort study*—Summarise follow-up time (eg, average and total amount) |  |  |
| Outcome data | 15* | *Cohort study*—Report numbers of outcome events or summary measures over time |  |  |
|  |  | *Case-control study—*Report numbers in each exposure category, or summary measures of exposure |  |  |
|  |  | *Cross-sectional study—*Report numbers of outcome events or summary measures | *6* | are reported using tables and graphs |
| Main results | 16 | (*a*) Give unadjusted estimates and, if applicable, confounder-adjusted estimates and their precision (eg, 95% confidence interval). Make clear which confounders were adjusted for and why they were included | 6 | In our linear regression models, the adjustment variables are indicated |
|  |  | (*b*) Report category boundaries when continuous variables were categorized |  |  |
|  |  | © If relevant, consider translating estimates of relative risk into absolute risk for a meaningful time period |  |  |

Continued on next page

| Other analyses | 17 | Report other analyses done—eg analyses of subgroups and interactions, and sensitivity analyses | 6 |  |
| --- | --- | --- | --- | --- |
| Discussion | | | | |
| Key results | 18 | Summarise key results with reference to study objectives | 6-7 | “Our results indicate that nutritional status, specifically BMI and fat mass classification, is related to muscle reaction time in children.” |
| Limitations | 19 | Discuss limitations of the study, taking into account sources of potential bias or imprecision. Discuss both direction and magnitude of any potential bias | 8 | “Among this study's limitations are the non-probabilistic participant selection and the small sample size. The latter hindered the classification of children into more nutritional status categories, such as average weight, overweight, or obese, and instead required a binary categorization. Another limitation of the study was the lack of assessment of subcutaneous fat, which can on impact the acquisition of sEMG signals by acting as a low-pass filter.” |
| Interpretation | 20 | Give a cautious overall interpretation of results considering objectives, limitations, multiplicity of analyses, results from similar studies, and other relevant evidence | 7-8 | develops throughout the discussion |
| Generalisability | 21 | Discuss the generalisability (external validity) of the study results | 7-8 | develops throughout the discussion |
| Other information | |  | | |
| Funding | 22 | Give the source of funding and the role of the funders for the present study and, if applicable, for the original study on which the present article is based | 9 | “This research was financed by the internal funds of the Academic Vice-Rector for Research and Postgraduate Studies of the Universidad Santo Tomás (Chile) (project number 113200024).” |

*Give information separately for cases and controls in case-control studies and, if applicable, for exposed and unexposed groups in cohort and cross-sectional studies.

**Note:** An Explanation and Elaboration article discusses each checklist item and gives methodological background and published examples of transparent reporting. The STROBE checklist is best used in conjunction with this article (freely available on the Web sites of PLoS Medicine at http://www.plosmedicine.org/, Annals of Internal Medicine at http://www.annals.org/, and Epidemiology at http://www.epidem.com/). Information on the STROBE Initiative is available at www.strobe-statement.org.
